# AAV-mediated gene transfer to colon-innervating primary afferent neurons

**DOI:** 10.3389/fpain.2023.1225246

**Published:** 2023-08-04

**Authors:** Reshma Gore, Tina Esmail, Kelsey Pflepsen, Ezequiel Marron Fernandez de Velasco, Kelley F. Kitto, Maureen S. Riedl, Andrea Karlen, R. Scott McIvor, Christopher N. Honda, Carolyn A. Fairbanks, Lucy Vulchanova

**Affiliations:** ^1^ Department of Neuroscience, University of Minnesota, Minneapolis, MN, United States; ^2^ Department of Pharmaceutics, University of Minnesota, Minneapolis, MN, United States; ^3^ Department of Pharmacology, University of Minnesota, Minneapolis, MN, United States; ^4^ Department of Genetics, Cell Biology and Development, University of Minnesota, Minneapolis, MN, United States

**Keywords:** AAV, visceral, nociception, chemogenetic, intrathecal, colon, sensory

## Abstract

Investigation of neural circuits underlying visceral pain is hampered by the difficulty in achieving selective manipulations of individual circuit components. In this study, we adapted a dual AAV approach, used for projection-specific transgene expression in the CNS, to explore the potential for targeted delivery of transgenes to primary afferent neurons innervating visceral organs. Focusing on the extrinsic sensory innervation of the mouse colon, we first characterized the extent of dual transduction following intrathecal delivery of one AAV9 vector and intracolonic delivery of a second AAV9 vector. We found that if the two AAV9 vectors were delivered one week apart, dorsal root ganglion (DRG) neuron transduction by the second vector was greatly diminished. Following delivery of the two viruses on the same day, we observed colocalization of the transgenes in DRG neurons, indicating dual transduction. Next, we delivered intrathecally an AAV9 vector encoding the inhibitory chemogenetic actuator hM4D(Gi) in a Cre-recombinase dependent manner, and on the same day injected an AAV9 vector carrying Cre-recombinase in the colon. DRG expression of hM4D(Gi) was demonstrated at the mRNA and protein level. However, we were unable to demonstrate selective inhibition of visceral nociception following hM4D(Gi) activation. Taken together, these results establish a foundation for development of strategies for targeted transduction of primary afferent neurons for neuromodulation of peripheral neural circuits.

## 1. Introduction

Chronic visceral pain is debilitating and often diffuse, poorly localized, and idiopathic. It may affect multiple organs at once and is both difficult to study and treat. A major challenge in investigating the circuits underlying chronic visceral pain is the neuroanatomical complexity of the peripheral nervous system. The innervation of peripheral organs is comprised of complex neural circuits supplied by multiple nerves that commonly carry both efferent and afferent axons and target multiple organs. The functional analysis of peripheral neural circuits would be facilitated by selective targeting of neuromodulatory genes to afferent or efferent peripheral neurons using adeno-associated viral (AAV) vector-mediated gene transfer. In addition to aiding the investigation of visceral pain mechanisms, AAV strategies that allow for neuromodulation of primary afferent neurons without affecting peripheral autonomic neurons have the potential to inform the development of gene therapy-based treatments.

In the gastrointestinal tract, the complexity of peripheral organ innervation is compounded by the intrinsic neural circuits of the enteric nervous system. The extrinsic sensory innervation of the colon is supplied by primary afferent neurons that reside in thoracolumbar and lumbosacral dorsal root ganglia (DRG) and transmit sensory information from the colon to the spinal cord under normal conditions and under conditions of visceral hypersensitivity. We previously demonstrated that intracolonic injection of AAV vectors leads to transgene expression in both DRG neurons and enteric neurons (1). In this study, we explored the feasibility of a dual-vector strategy that targets transgenes specifically to colon-innervating sensory neurons. The dual-vector strategy, used extensively for projection-specific transgene expression and neuroanatomical and functional circuit tracing in the CNS (2), employs an AAV carrying a Cre-dependent transgene of interest and a second AAV carrying Cre-recombinase, delivered at the levels of the neuronal cell body and synaptic terminals, respectively. We adapted this approach by delivering one of the vectors intrathecally (i.t.) at the level of the central processes and cell bodies of DRG neurons and the other vector intracolonically (i.c.) at the level of the peripheral processes of colon-innervating DRG neurons. Cre-dependent expression of the inhibitory DREADD (designer receptors exclusively activated by designer drugs) hM4D(Gi) was employed to attenuate visceral nociception induced by intracolonic capsaicin (3).

## 2. Materials and methods

### 2.1. Animals

All procedures were approved by the Institutional Animal Care and Use Committee at the University of Minnesota and accordance with the National Institutes of Health *Guide for Care and Use of Laboratory Animals*. Male and female CD-1 (ICR) mice, purchased from Envigo (Indianapolis, IN) or bred in-house, were used for all experiments. Animals were group housed in a specific pathogen-free rodent vivarium at the University of Minnesota.

### 2.2. Viral vectors

rAAV9/CAG-tdTomato (5.9 × 10^12 ^vg/ml), abbreviated AAV9-tdT, from UNC Gene Therapy Vector Core, Chapel Hill, NC; AAV9-hSyn-CreGFP (2.7 × 10^13 ^vg/ml), abbreviated AAV9-CreGFP, from Penn Vector Core, University of Pennsylvania, PA; AAV9-hSyn-DIO-hM4D(Gi)-mCherry (6.12 × 10^12 ^vg/ml), abbreviated AAV9- hM4Di, from Penn Vector Core, University of Pennsylvania.

### 2.3. Injections and surgical procedures

**Figure 1:**
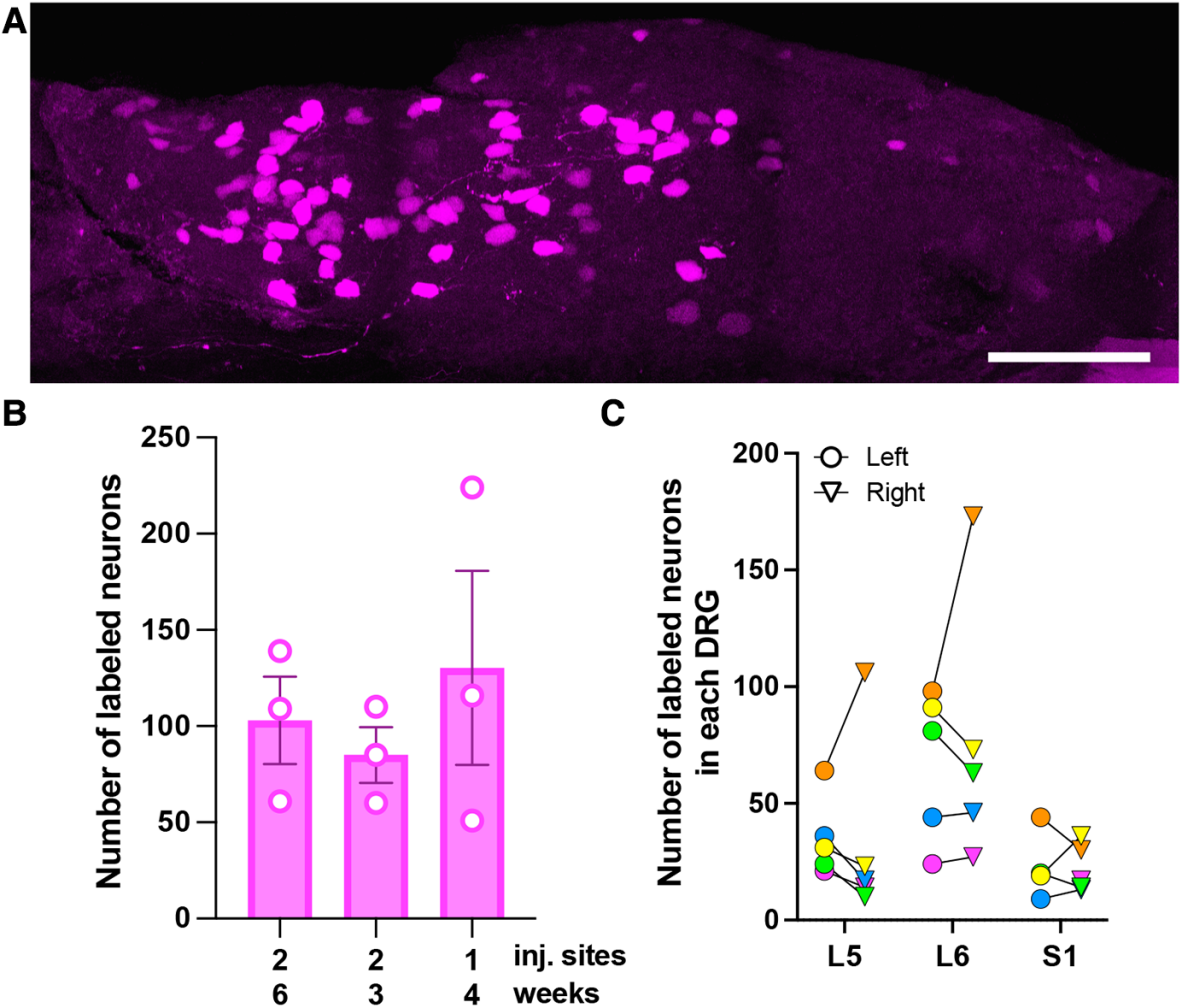
Characterization of AAV9-mediated transduction of colon-innervating DRG neurons following i.c. delivery. (**A**) A representative image of a whole-mounted L6 DRG. The image is a maximum intensity projection of 47 optical sections, 2 μm z-step. Scale bar: 200 μm. (**B**) Comparison of the number of tdTomato-labeled neurons in L5-S1 DRG with one or two i.c. injection sites and 3-, 4-, or 6-week survival times. Each data point represents the sum of labeled neurons in L5-S1 ganglia, including only the left or right ganglion for each level (*n* = 3 male mice per condition; *p* = 0.39, one-way ANOVA). (**C**) Bilateral distribution of tdTomato-labeled neurons in L5-S1 DRG (*n* = 5; 2 males—green and yellow symbols, and 3 females—orange, pink and blue symbols). Circles and triangles indicate left and right ganglia, respectively.

**Figure 2:**
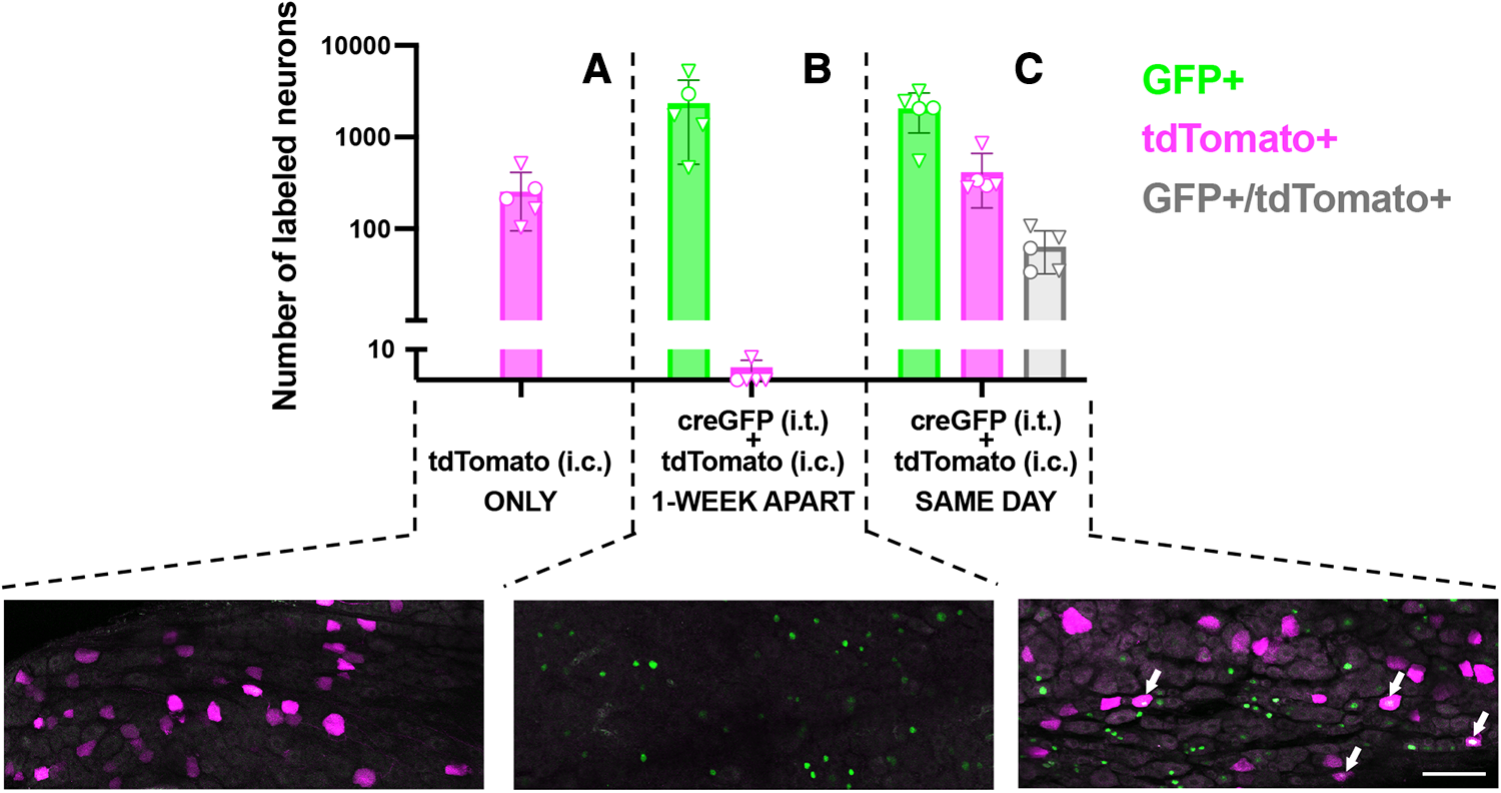
Quantitative analysis and representative images of transduction of colon-innervating DRG neurons following dual AAV delivery. (**A**) tdTomato labeling in DRG neurons in mice that received an intrathecal injection of saline and an intracolonic injection of AAV9-tdT (*n* = 5, 2 males and 3 females, identified by circles and triangles, respectively). (**B**) Nuclear CreGFP labeling in DRG neurons in mice that received an intrathecal injection of AAV-CreGFP and a week later received an intracolonic injection of AAV9-tdT (*n* = 5, 1 male and 4 females, identified by circles and triangles, respectively). tdTomato labeling in these DRG was negligible. GFP labeling is restricted to the nucleus due to the nuclear targeting of the CreGFP fusion protein. (**C**) Mice received an intrathecal injection of AAV-CreGFP and an intracolonic injection of AAV9-tdT on the same day (*n* = 5, 2 males and 3 females, identified by circles and triangles, respectively). Arrows indicate neurons that express both CreGFP and tdTomato. All images are a maximum intensity projection of 6 optical sections, 2 μm z-step. Scale bar: 100 μm.

**Figure 3:**
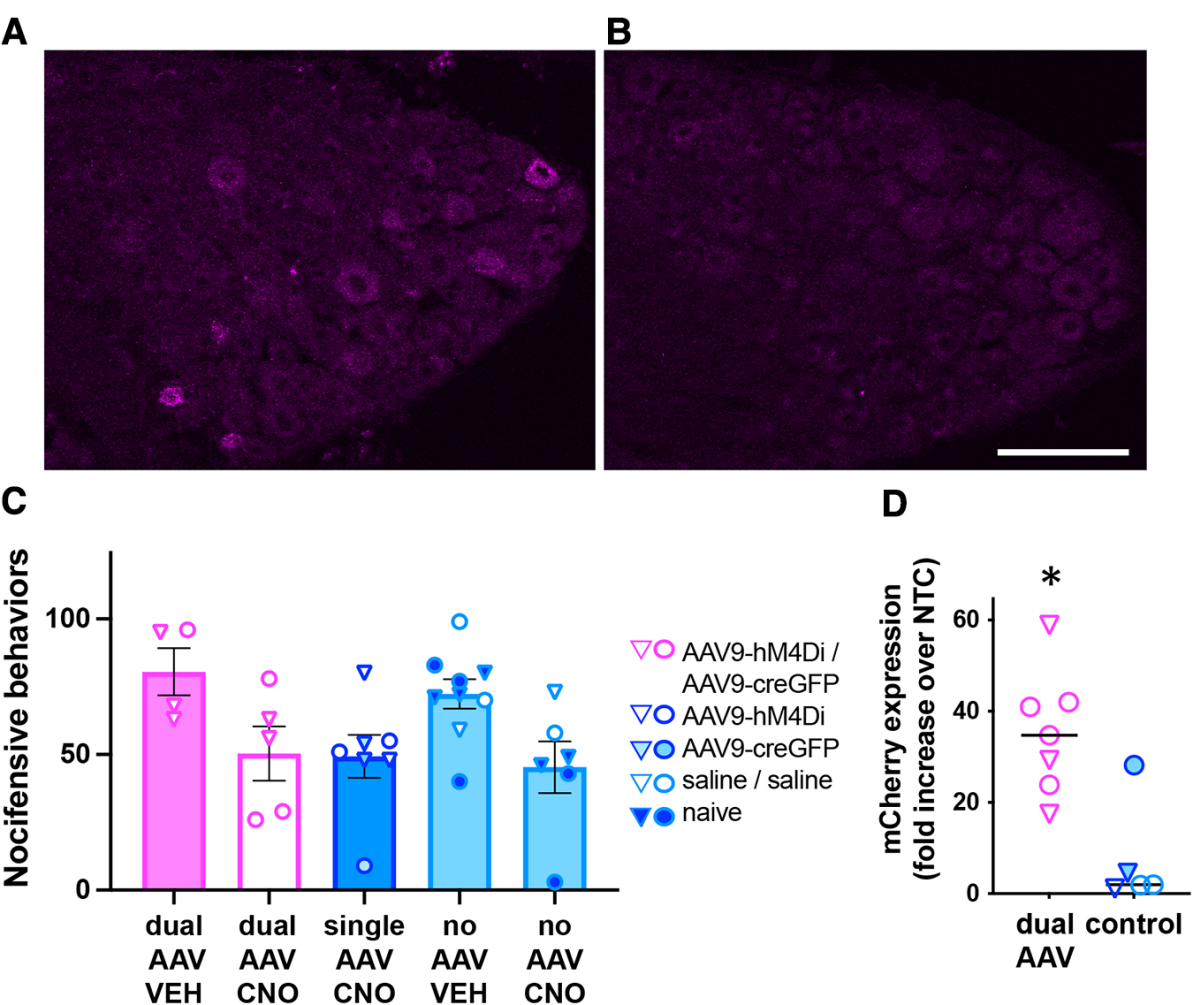
Analysis of dual-AAV-mediated Cre-dependent transgene expression in DRG. (**A**,**B**) Immunolabeling for mCherry demonstrated expression of the hM4D(Gi)-mCherry transgene in L6 DRG from mice injected with AAV9- hM4Di (i.t.) followed by same-day intracolonic injection of AAV9-CreGFP (**A**) or Saline (**B**). Scale bar: 100 μm. (**C**) Behavioral analysis of CNO-mediated inhibition (10 mg/kg, i.p.) of nocifensive behaviors induced by intracolonic capsaicin. Triangles and circles indicate female and male subjects, respectively; *n* = 4−9 per group. (**D**) RT-qPCR analysis of mCherry expression in L5-S1 DRG from a subset of the animals used for the behavioral experiment in C. The mCherry expression levels were significantly higher in dual-AAV injected animals compared to animals that received control treatments (unpaired *t*-test, *p* < 0.005). Expression levels were normalized to the no template control (NTC, please see methods). The symbol legend is the same as in (**C**).

Intrathecal (i.t.) injections (10 μl of viral vector) were made by inserting a 30-gauge needle between the L3-L4 vertebrae of awake 3–4-week old mice (4–6). Intracolonic (i.c.) injections were performed on the same day or a week following i.t. injections as described (1). Briefly, under isoflurane (2.5%–4%) anesthesia, the descending colon was exposed following a vertical incision (∼3 cm) in the lower abdomen. Mice received a single 4 μl injection of viral vector (with the exception of one experiment in which numbers of transduced DRG neurons following one or two injections were compared). Injections were made under the serosal layer of the colon wall, approximately 2 cm from the anal verge, using a 10 μl Hamilton syringe attached to a 30-gauge needle. The needle was left in place for 1 min to prevent reflux. Following injection, the abdominal wall was sutured (Ethicon Cat# Z304H), and the overlying skin was closed with Vetbond. Mice were allowed to recover on a heated pad and meloxicam (2 mg/kg, s.c.) was given for post-operative analgesia for three consecutive days. Tissues were collected for histological analysis 3–6 weeks after colon injections (Figures 1–3A,B).

### 2.4. Histological analysis

#### 2.4.1. Tissue collection and preparation

Mice were deeply anesthetized with isoflurane and perfused via the heart with calcium-free Tyrode’s solution (in mM: 116 NaCl, 5.4 KCl, 1.6 MgCl_2_^·^6H_2_0, 0.4 MgSO_4_·7H_2_O, 1.4 NaH_2_PO_4_, 5.6 glucose, and 26 Na_2_HCO_3_) followed by fixative (4% paraformaldehyde and 0.2% picric acid in 0.1 M phosphate buffer, pH 6.9). Bilateral DRG were collected and prepared as cryostat sections or whole mounts. DRG cryostat sections: DRG were stored in 10% sucrose (in PBS) overnight, mounted using OCT (Tissue-Tek 4583), frozen with liquid CO_2_, and cryostat-sectioned into 14-μm sections that were mounted on gelatin-coated slides and stored at −20°C. DRG whole mounts: Following dissection, DRG were stored in PBS at 4°C. DRG were then mounted on gelatin-coated slides and coverslipped using FluorSave (Millipore cat#345789) mounting media.

#### 2.4.2. Immunohistochemistry

Thawed DRG sections were incubated in a blocking buffer (PBS containing 0.3% Triton-X 100, 1% BSA, 1% normal donkey serum) for 30 min followed by overnight incubation in primary antibody solution (Rabbit anti-DsRed (1:1,000), Living Colors®; Chicken anti-GFP(1:1,000), Abcam #13970) at 4°C. Following three 10-min washes in PBS, slides were incubated with secondary antibody solution (Cy3 Donkey anti-Rabbit 1:600, Alexa488 Donkey anti-Chicken 1:100, Jackson labs) for 1 h at RT, washed again with PBS (3 × 10 min), and stained with NeuroTrace (1:1,000, Invitrogen #N21479). Coverslips were placed onto the slides using FluorSave mounting media.

#### 2.4.3. Imaging and quantification

DRG sections were imaged using an Olympus BX2 microscope equipped with a Fluoview 1,000 scan head, software version 4.1.5.5; objective—UPLSAPO 20x/0.85 NA, using uniform imaging parameters that avoided saturation. Image analysis was performed by trained observers masked to the experimental groups using Fiji as previously described (7). Neuronal profiles with nuclei were identified based on NeuroTrace labeling and outlined in 5 evenly spaced sections per DRG. Measurements of the area and the mean grey value of tdTomato immunoreactivity were obtained for each profile. The number of tdTomato + and GFP + cells were manually counted. The data are reported as the percentage of labeled neurons out of all outlined profiles.

Native tdTomato and GFP fluorescence was imaged in whole-mounted DRG with a Nikon FN1 upright stand equipped with an A1R HD MP laser scanning head and a motorized Prior stage and piezo Z drive (for sample positioning and focus) and controlled with NIS Elements 5.1 software; objective—Nikon Plan Apo LWD 25 × water-immersion/NA 1.1 (multiphoton mode, Mai Tai DeepSee 920 nm excitation, 525–595 nm emission, GaAsP NDD). Stitched 3D stacks of raw images spanning the entire ganglion (collected with a 2 or 3 μm z-step) were evaluated by an observer masked to the experimental conditions to identify and count tdTomato + and GFP + cells. Unlabeled neurons were not counted. The data are reported as numbers of labeled neurons (not percentages). Representative images were adjusted for brightness and contrast, with uniform adjustments across experimental groups.

## 2.5. Behavioral analysis

4–6 weeks after the intracolonic vector injections, animals were acclimated to the behavior assessment room for 1 h, followed by intraperitoneal injections of Clozapine-N-Oxide (CNO) or saline solution (10 mg/kg; HelloBio cat#HB1807). Twenty minutes after the CNO or saline injections, a PE50 tubing was inserted 2 cm into the anus and 10 μl of 0.6% capsaicin (in sterile saline) solution was injected. The tubing was held in place for 30 s to prevent capsaicin backflow and avoid capsaicin exposure to surrounding tissue. Mice were then placed in transparent glass chambers containing bedding material and video recorded for 20 min. Nocifensive behaviors (scratching, writhing, licking) were counted by two observers masked to treatment conditions, and their results were averaged to obtain counts of behaviors suggesting visceral nociception.

## 2.6. Reverse-transcriptase quantitative PCR (RT-qPCR) analysis

L5-S1 DRG were dissected from a subset of the mice used for behavioral testing. Dissected DRG were homogenized in 400 μl of DNA/RNA Shield (ZymoResearch) using one scoop of 0.5 mm glass beads (BioSpec Products) and a Bullet Blender (Next Advance). RNA was extracted and purified (Quick-RNA miniprep kit, ZymoResearch), and then dissolved in nuclease-free water. RNA concentration was determined using the NanoDrop ND-1000 spectrophotometer (Thermo Fisher Scientific). The expression of hM4D(Gi)-mCherry was determined by RT-qPCR, using a CFX96 Touch Real-Time PCR Detection System (Bio-Rad), the iTaq™ Universal SYBR® Green One-Step Kit (Bio-Rad), and mCherry primers (F: 5′-GAA CGG CCA CGA GTT CGA GA-3′ and R: 5′-CTT GGA GCC GTA CAT GAA CTG AGG-3′). Each 20 μl reaction mixture contained 10 μl of 2X iTaq™ Universal SYBR® Green Reaction Mix, 5.75 μl of RNase/DNase free H_2_O, 0.25 μl iScript™ Reverse Transcriptase, 80 nM each of forward and reverse primer, and 40 ng of RNA sample. All samples were run in triplicate. Two no-template control (NTC) reactions and one RNase/DNase free H_2_O (20 μl each) blank were included in each run. PCR cycling conditions: 20 min at 50°C and 1 min at 95°C, then 35 cycles each of 15s denaturation at 95°C and 30s annealing and extension at 55.8°C. Melting curves were analyzed to ensure a single PCR product for each reaction. PCR product concentrations were interpolated from the C_T_ values of serially diluted (10^12^–10^6^) AAV.hsyn.DIO.hM4D(Gi)-mCherry.WPRE.hGH plasmids of a known concentration containing the mCherry sequence (6.12 × 10^12^ vg/ml). The data were normalized to the NTC by dividing the resulting copy number for each sample by the copy number detected in the NTC.

## 2.7. Statistical analysis

All statistical analyses were performed using GraphPad Prism 9.4.1. Neuroanatomical data were analyzed using Student’s *t*-test for comparisons of two groups or one-way ANOVA and Tukey’s post-hoc test for comparisons of three conditions. Behavioral data were analyzed using two-way ANOVA and Tukey’s post-hoc test. RT-qPCR data were analyzed using Student’s *t*-test. Although group sizes were not statistically large enough to compare viral transduction and capsaicin-induced nociception in male and female mice, the data did not reveal trends suggestive of sex differences.

## 3. Results

### 3.1. Characterization of AAV9-mediated transduction of colon-innervating DRG neurons following i.c. delivery

To optimize viral transduction after i.c. delivery, we injected AAV9-tdT (4 μl) at one or two locations in the colon and collected tissues 3 (two injections), 4 (one injection), and 6 (two injections) weeks later. Quantitative analysis was performed in whole-mount DRG preparations (Figure 1A). We did not observe significant differences in the number of tdT labeled cells in the L5-S1 DRG under the different conditions [Figure 1B; one-way ANOVA, F(2,6) = 0.48, *p* = 0.64]. In all subsequent experiments, we performed a single 4 μl injection and collected tissue 3–6-weeks post-injection. Quantitative analysis of tdT-expressing cells in whole-mounted T10-S1 DRG demonstrated that the lumbosacral L5-S1 DRG contained more transduced neurons than thoracolumbar T10-L1 DRG (108 ± 42 for L5-S1 vs. 38 ± 11 for T10-L1; *n* = 4 male mice). Some labeled neurons were also present in L2-L4 DRG. Subsequent analyses were focused on lumbosacral DRG. The total number of tdT-labeled neurons in left and right L5-S1 ganglia, quantified in whole-mounted DRG, was 254 ± 71. Variability in the number of transduced neurons was noted between animals and between left and right L5-S1 DRG within individual animals (Figure 1C). Using quantitative image analysis of DRG cryostat sections, we estimated that 2.4 ± 0.6% of L5-S1 DRG neurons were tdT-positive following i.c. AAV9-tdT delivery.

### 3.2 Characterization of dual AAV transduction in lumbosacral DRG

To evaluate the potential for dual-vector targeting of colon-innervating neurons, we examined the extent of co-expression of CreGFP and tdT (non-Cre-dependent) following i.t. and i.c. injections, in the same animal. For logistical reasons, the i.t. injection of AAV9-CreGFP or saline was performed a week prior to the i.c. injection of AAV9-tdT. Quantitative analysis of GFP labeling in DRG cryostat sections indicated that 30 ± 10% of L5-S1 neurons were GFP-positive. Surprisingly, analysis of tdT labeling indicated that the proportion of tdT-expressing neurons was significantly lower in the group that received i.t. injection of AAV9-CreGFP compared to the group that received i.t. injection of saline (2.4 ± 0.6% vs. 0.4 ± 0.2%, *p* < 0.05, unpaired *t*-test; *n* = 5, 3 males and 2 females per group). To test whether the 1-week delay between the i.t. and i.c. injections caused the low expression of tdT, we compared tdT labeling in mice that received the two injections 1-week apart to mice injected with the two vectors on the same day (Figure 2; analysis of whole-mounted DRG). When the AAV9-CreGFP (i.t.) was injected a few hours prior to AAV9-tdT (i.c.), we detected a significantly higher number of tdT-positive cells compared to the 1-week apart condition [Figure 2; *p* < 0.01, One-way ANOVA, *F*(2,12) = 7.6, Tukey’s multiple comparison test]. Analysis of the colocalization of GFP and tdT labeling indicated that approximately 16% (range 11%–26%) of tdT-expressing colon-innervating DRG neurons were GFP-positive (Figure 2).

### 3.3 Analysis of dual-AAV-mediated Cre-dependent transgene expression in DRG

We next evaluated whether same-day delivery of Cre-dependent AAV9-hM4Di (i.t.) and AAV9-CreGFP (i.c.) resulted in DRG expression of the neuromodulatory gene hM4D(Gi)-mCherry, whose activation mediates inhibition of neural activity (8). Expression of hM4D(Gi)-mCherry was visualized by mCherry immunolabeling (Figures 3A,B), but low signal-to-noise ratio precluded rigorous quantitative estimate of the proportion of labeled neurons. In a separate cohort of mice, we then conducted a behavioral experiment to determine whether activation of hM4Di with the ligand CNO reduces nocifensive behaviors elicited by intracolonic capsaicin injection. Pilot testing of CNO (10 mg/kg, i.p.) did not reveal non-hM4D(Gi) effects. However, statistical analysis of our behavioral data following dual AAV and control treatments indicated an effect of CNO regardless of viral treatment, suggesting non-hM4Di mediated inhibition of nocifensive behaviors by CNO (Figure 3C; two-way ANOVA; *p* < 0.005, CNO vs. vehicle). RT-qPCR analysis in L5-S1 DRG of a subset of these mice confirmed the expression of hM4D(Gi)-mCherry following dual-vector treatment (Figure 3D; *p* < 0.05, unpaired *t*-test). Therefore, although we did not detect a behavioral effect of hM4Di-mCherry, we confirmed that the neuromodulatory transgene was indeed expressed.

## 4. Discussion

While AAV vector approaches for neuronal gene transfer have enabled intricate circuit mapping in the CNS (9), their application in the peripheral nervous system (PNS) has been limited (10–13). The present study explored the feasibility of organ-selective dual AAV targeting of peripheral neurons. Compared to the use of a single retrograde virus, this strategy has the potential to enable expression of neuromodulatory genes in primary afferent neurons without expression in postganglionic autonomic neurons. Alternative strategies for selective targeting of afferent and efferent peripheral neurons, such as AAV constructs with cell-specific promoters or serotypes with differential tropism are not readily available at present. This investigation was focused on targeting gene transfer to colon-innervating DRG neurons using dual i.t. and i.c. AAV delivery. Our results demonstrated successful Cre-dependent transgene expression in DRG neurons following i.t. delivery of AAV9 carrying a Cre-dependent hM4Di-mCherry and i.c. delivery of AAV9 carrying Cre-recombinase. While we achieved proof-of-concept for dual gene transfer to primary afferent neurons via centrally and peripherally administered viruses, this work revealed the need for additional optimization of this approach.

The choice of AAV9 for these experiments was motivated by the relatively high neuronal tropism of this serotype (5, 7, 14). Comparisons of the numbers of colon-innervating primary afferent neurons labeled in this study and in previous reports are difficult due to different methodologies. Our estimate of the proportion of labeled neurons in L5-S1 DRG is lower than the proportion reported following i.c. injection of Cre-dependent AAV9 in TRPV1-Cre mice (15). However, our counts of labeled lumbosacral neurons in whole-mounts are consistent with results from previous studies, in which cholera toxin B (CTB) was injected into the colon and transported to DRG cell bodies (16, 17). We also noted some transduced neurons in L2-L4 DRG. Since the number of CTB-labeled neurons in these ganglia was reported to be negligible (16), the transduction we observed could be the result of limited systemic redistribution of AAV9 following i.c. delivery, which we previously described (7). Transduction of colon-innervating primary afferent neurons could be further optimized by delivery of smaller injection volumes in multiple locations (15) or the use of other AAV serotypes with less efficient systemic distribution (18).

An unexpected observation in the present study was the dependence of dual transduction on the timing between the i.t. and i.c. AAV9 delivery. The observation that i.t. delivery of AAV9 severely limits transduction by an i.c. AAV9 injection administered a week later suggests activation of host defense mechanisms by i.t AAV9. Systemic redistribution following i.t. AAV9 delivery has been documented and could induce activation of innate and adaptive immune responses (5, 19). However, if transduction of colon innervating sensory neurons by i.c. delivery relies on viral vector uptake via their peripheral terminals, it should not be greatly impacted by AAV neutralization by systemic immunity. Alternatively, host-defense mechanisms activated within DRG following the initial exposure to AAV9 may downregulate the cell surface receptors that mediate neuronal AAV9 entry, although to our knowledge such AAV-dependent downregulation has not been documented in the literature. Potential host-defense mechanisms could be mediated by non-neuronal DRG cells as AAV particles have been observed in association with myeloid cells within the DRG 30 min after i.t. viral delivery (20). Engagement of neuronal cellular defense mechanisms, such as activation of pattern recognition receptors or cellular stress response pathways, is also possible (21–24). It is unclear if the observed effects of initial AAV exposure were specific to the AAV9 serotype and if the use of a different serotype for the i.c. injections [e.g., AAV6 (25)] could circumvent the necessity to deliver the vectors on the same day.

Following i.t. AAV9-CreGFP and i.c. AAV9-tdT delivery on the same day, we observed colocalization of CreGFP and tdTomato labeling in DRG neurons, demonstrating successful dual transduction by the centrally and peripherally administered viruses. Quantitative analysis of the colocalization indicated that GFP fluorescence was detected only in 16% of tdTomato-expressing neurons. Based on our estimates of the proportions of CreGFP-expressing L5-S1 neurons following i.t. delivery (∼30%) and tdT-expressing neurons following intracolonic delivery (∼2.4%), this relatively low incidence of colocalization is not surprising. However, the extent of dual transgene expression may be underestimated, as we have previously observed that detection of CreGFP may be limited by simultaneous expression of tdTomato (7). In future studies, the efficiency of dual transduction may be enhanced if i.c. delivery is combined with direct AAV injection in DRG (26–28).

Successful dual AAV targeting of colon-innervating DRG neurons was further ascertained by the detection of Cre-dependent expression of hM4Di. However, we were unable to demonstrate functional effects of hM4Di through the inhibition of capsaicin-induced nocifensive behavior. This may be due to a combination of factors, including low efficiency of dual transduction, non-hM4Di-mediated effects of CNO, low sample size, and the high variability in the behavioral responses to intracolonic capsaicin, which may have obscured the detection of these non-specific effects in our pilot studies. Although a range of CNO doses has been used in previous studies (8, 15, 28, 29), the rationale for selecting a higher dose was that our neuroanatomical experiments suggested a relatively low dual transduction efficiency. Newly developed DREADD ligands with high *in vivo* potency may facilitate the application of chemogenetic approaches in the peripheral nervous system (30).

We have previously reported the transduction of enteric neurons following i.c. delivery (1) and some systemic redistribution of AAV9 following both i.t. and i.c. delivery (5, 7). The present study did not evaluate potential off-target Cre-dependent recombination in the enteric nervous system or other peripheral organs that could result from access of AAV9 to systemic circulation. However, the potential for such off-target effects could be eliminated in future studies by refinement of the site-specific delivery of the two viruses and optimization of AAV serotypes. Further development of dual AAV Cre-dependent gene transfer to colon-innervating DRG neurons will enable their manipulation without affecting intrinsic enteric neurons. Such selective manipulation is currently hindered by the extensive overlap of gene expression between primary afferent and enteric neurons. For example, TRPV1 is expressed by the majority of colon-innervating DRG neurons and by some enteric neurons (31, 32).

In conclusion, we have demonstrated the feasibility of organ-selective dual AAV targeting of primary afferent neurons via central and peripheral viral vector delivery. Optimization of this approach has the potential to facilitate the investigation of mechanisms of visceral pain and interoception by allowing manipulation of primary afferent neurons without affecting efferent peripheral neurons and beyond the constraints of transgenic mouse lines. Furthermore, extending dual AAV targeting to a combination of peripheral delivery of transsynaptic AAV to primary afferent neurons and intraspinal viral delivery has the potential to enable organ-selective exploration of spinal circuits of visceral pain (33).

## Data Availability

The raw data supporting the conclusions of this article will be made available by the authors, without undue reservation.
